# Assessing Paracetamol Overdose in Children: Acceptability and Potential Market for a Non-Invasive Testing Device

**DOI:** 10.1177/11795972221140108

**Published:** 2023-01-31

**Authors:** Debora Freitas, Christopher Parry, Gabrielle Seddon, Jana Lemke, James Moss, Neville Freeman, Julie Grice, Daniel B Hawcutt

**Affiliations:** 1 Emergency Department, Alder Hey Children’s Hospital, Liverpool, UK; 2 NIHR Alder Hey Clinical Research Facility, Liverpool, UK; 3 Department of Women’s and Children’s Health, Institute of Life Course and Medical Sciences, University of Liverpool, Liverpool, UK; 4 Paediatric Medicines Research Unit, Alder Hey Children’s Hospital, Liverpool, UK; 5 University of Greifswald, Greifswald, Germany; 6 Nanoflex Ltd, Warrington, UK; 7 E4G Ltd, Neston, UK

**Keywords:** Paediatric, paracetamol, overdose, therapeutic drug monitoring, wearables

## Abstract

**Background:**

Assessment of paracetamol overdose in children and teenagers in the emergency department (ED) requires blood, taken 4 hours post ingestion. A commercial partner developed transdermal paracetamol measuring technology. This work aims to understand the acceptability of such a device, and potential market size.

**Methods:**

A questionnaire study was undertaken with children and parents attending Alder Hey Children’s Hospital, and healthcare professionals (HCP) involved in their care. A retrospective audit of paracetamol ingestion presenting to a paediatric ED was undertaken.

**Results:**

One hundred forty-three questionnaires were distributed, and 139 returned (response rate 97.2%), comprising 55 children, 52 parents and 32 HCP (recruited between August-October 2019). Overall device acceptability, assessed by favourability of appearance and willingness to wear was high, at 60.0% and 81.5% respectively. Concerns raised included bulky size and weight, and concern regarding the duration younger children would tolerate wearing the device. All groups, including children, ranked accuracy of results as the most important device feature and device comfort the least important. Parents prioritised avoidance of blood tests more than children. One hundred twenty-seven children presented to ED with paracetamol ingestion (September 2017-August 2018), with 57 (44.9%) categorised as accidental overdose. Overall, 106 (83.4%) required paracetamol concentration measuring, and 25 (19.7%) of these required treatment with N-acetylcysteine. Extrapolating nationally, over 7000 children will present with accidental overdose per annum in the UK.

**Conclusion:**

Acceptability of a non-invasive paracetamol sensor was high in all groups, provided accuracy could be assured.

## Introduction

Paracetamol (acetaminophen) is one of the most widely administered medicines in children, commonly used for fever and pain. The National Institute for Health and Care Excellence (NICE) guidance recommend paracetamol as an anti-pyretic in children under 5 years who appear distressed during a febrile illness.^1^ The UK Health Security Agency (previously Public Health England) also advise parents to give children paracetamol when given the meningitis B vaccine at 2 and 4 months of age, as part of the UK immunisation schedule.^2^ Calpol™, the main brand of paracetamol suspension for children in the UK, advertises paracetamol suspension as ‘gentle on delicate tummies’^3^ and ‘trusted by parents for over 50 years’.^3^ As such paracetamol is likely to be perceived as a safe medication to give to children by parents. By the age of 6 months up to 84% of children in the UK will have received paracetamol.^4^

Paracetamol is also the most common drug taken in overdose in the UK with approximately 100 000 patients presenting to emergency departments (ED) each year.^5^ Children and teenagers (defined as up until the day before their 18th birthday) form a significant percentage of this cohort. For brevity, children and teenagers will be referred to as ‘children’. Overdoses of paracetamol in paediatrics can be divided into 2 main categories: accidental overdose (mainly associated with toddlers) and non-accidental overdoses (mainly associated with teenagers). Fifty children between ages of 0 and 18 years died of suicide or deliberate self-inflicted harm in the UK in 2010; this number doubled to 101 by 2017.^6^

Clinicians managing paediatric paracetamol overdoses can be faced with a number of challenges including uncertainty of the ingested dose, delayed presentation, or staggered overdose. A single paracetamol dose of >150 mg/kg may cause hepatocellular damage.^7^In cases where there is uncertainty over the dose ingested clinicians rely on plasma paracetamol concentration at 4 hours post ingestion.^8^ However, in children the process of venepuncture can be a painful, distressing and frightening experience.^9,10^ The timing of a sample 4 hours after ingestion also means that those presenting promptly often have to wait a long time in ED.

If an individual presenting to the ED could be screened for a potentially hepatotoxic paracetamol overdose using a non-invasive method, then if low/zero concentrations are noted, discharge from ED would be facilitated and patient experience improved, as the standard blood test 4 hours post-ingestion could potentially be avoided. In addition, if the non-invasive test suggests a high likelihood of toxicity, appropriate diagnostic tests can be ordered, and care strategies implemented.

A company have developed a device that could be worn by the patient post triage when a paracetamol overdose is suspected. The device consists of a disposable electrochemical sensor element which can determine serial measurements of paracetamol in sweat. The sensor has been designed to detect the direct, irreversible oxidation of paracetamol at the working electrode in the nanomolar range. The information gathered is then reported back to the emergency department to help guide decision making regarding the early stages of care and the need for diagnostic blood tests to be undertaken. Once the device has completed its measurement it can be removed, and the sensor element replaced ready for future use. Prior to continuing product development, the company wished to determine the acceptability of such a device in the UK, and potential market size.

The primary objective of this study was therefore to determine the acceptability of a wearable device to screen children who may present to the ED with a suspected overdose of paracetamol. Secondary objectives included determining opinions regarding the device design, accuracy and acceptable duration of monitoring. We also sought to determine the prevalence of children attending a paediatric ED with paracetamol ingestion.

## Methods

### Study design and setting

A prospective questionnaire-based study of children and parents who attended ED, outpatient clinic or an inpatient ward, or healthcare professionals working with patients at Alder Hey Children’s Hospital. Questionnaires provided to children were designed to be age-appropriate and split into 3 groups: 6 to 11 years, 12 to 15 years and 16 to 18 years, with design input from the Liverpool Young People’s Advisory Group. The study recruited from August to October 2019. The full questionnaires are shown in the Supplemental data section.

In addition, a retrospective audit was performed of all children attending ED at Alder Hey Children’s Hospital with paracetamol ingestion between September 2017 and August 2018. Data was collected from electronic health records using an electronic data collection form.

### Ethics approval

Was granted by NW Health Research Authority using the IRAS platform, REC Reference No: 19/WM/0158. The study was funded by the MRC Confidence in Concept scheme.

After provision of written and verbal information pertaining to the study, informed consent was implied for all participants who completed the relevant questionnaire. For children <16 years both the parent/guardian and the child were required to give implied consent. No participant identifiable data were collected.

### Inclusion criteria & exclusion criteria

#### Children

- Inclusion criteria:
  - Any child attending a tertiary level children’s hospital aged 0 to 18 years at time of recruitment who may require analgesia or anti-pyretic therapy
  - Good understanding of written and spoken English

- Exclusion criteria:
  - Age >18 years
  - Participant and parent/guardian unable to read and/or understand the study information sheet

#### Parent(s)/guardian(s)

- Inclusion criteria:
  - Relative of a child attending hospital who may require analgesia or anti-pyretic therapy

- Exclusion criteria
  - A healthcare professional

#### Healthcare professionals

- Inclusion criteria:
  - Provides healthcare to population of children in the UK

### Data handling and statistical analysis

All study data were compiled in a Microsoft Excel^™^ spreadsheet and stored securely as per General Data Protection Regulation (GDPR).

## Results

### Questionnaire

One hundred forty-three questionnaires were administered with a response rate of 97.2%. Table 1 shows the breakdown of the participants with 52 parents, 32 health care professionals (HCP) and 55 children. The HCP group comprised of nurses (13), doctors (10), health care assistants (4) and others (5).

**Table 1.** Breakdown of study participants.

Participant	Number, n	Percentage of total, %
6-11 y	20	14.4
12-15 y	20	14.4
16-18 y	15	10.8
Parent	52	37.4
HCP	32	23.0

**Figure 1.**
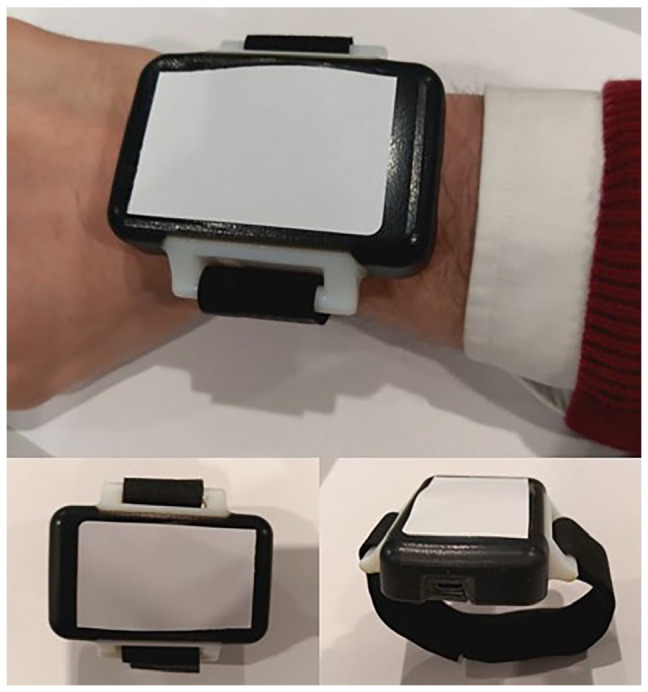
Prototype monitoring device, Nanoflex Ltd.

On being shown the prototype device (Figure 1), acceptability was high overall amongst children and parents, particularly in the 6 to 11 years old age group (Table 2). HCPs comparatively felt children would be less willing to wear the device. Participants were also asked how long they felt children would be willing to wear the device for. Answers varied vastly between groups, with younger people having no true preference and some participants in the teenager group willing to wear the device for 3 to 4 hours (Table 2).

**Table 2.** Device acceptability.

	6-11 y (n = 20)	12-15 y (n = 20)	16-18 y (n = 15)	Parents (n = 52)	HCP (n = 32)
Favourable appearance	13 (65%)	11 (55%)	*	*	*
Willingness to wear	19 (95%)	*	13 (87%)	44 (84%)	21 (65%)
Wearing duration (h)					
<0.5	5 (25%)	6 (30%)	2 (13%)	10 (19%)	N/A
0.5-1	1 (5%)	6 (30%)	4 (27%)	16 (31%)	N/A
1-2	4 (20%)	2 (10%)	4 (27%)	9 (17%)	N/A
2-3	5 (25%)	0 (0%)	0 (0%)	4 (8%)	N/A
3-4	5 (25%)	6 (30%)	5 (33%)	13 (25%)	N/A

Abbreviations: N/A, not applicable.

* Data not captured for this age group.

When asked about potential concerns or problems with wearing the illustrated prototype device HCPs had the most concern overall, particularly on whether children would be cooperative in wearing the device for the required length of time (Table 3). Comparatively children and parents placed heavier weight on the size of device, and felt a smaller device would be more appropriate.

**Table 3.** Concerns about wearing the device.

	6-11 y (n = 20)	12-15 y (n = 20)	16-18 y (n = 15)	Parents (n = 52)	HCP (n = 32)
Concerns about wearing device	3 (15%)	*	4 (27%)	13 (25%)	27 (84%)
Colour of device	0 (0%)	*	0 (0%)	1 (2%)	2 (6%)
Size of device	1 (5%)	*	4 (15%)	6 (12%)	6 (19%)
CYP cooperation	0 (0%)	*	0 (0%)	6 (12%)	17 (53%)
Other	2 (10%)	*	0 (0%)	0 (0%)	2 (6%)

* Data not captured for this age group.

Participants over the age of 12 years and parents were asked to rank the most important factor regarding a wearable device from a choice of 4. Only questionnaires without multiple nominations for first place were included. All groups ranked the most important factor as accuracy and the least important, comfort (Table 4).

**Table 4.** Ranking importance of device feature.

	12-15 y (n = 14)	16-18 y (n = 11)	Parents (n = 33)
Avoiding blood test	2 (14%)	2 (18%)	9 (27%)
Comfort of device	0 (0%)	0 (0%)	1 (3%)
Accuracy of result	9 (64%)	7 (64%)	17 (52%)
Faster diagnosis	3 (22%)	2 (18%)	6 (18%)

Finally children 12 years and older, parents and HCP were asked if they would be willing to participate in a clinical trial of the device. Generally positive results were found in all groups with an average of 85% of participants willing to be involved.

### Audit

One hundred twenty-seven attendances with paracetamol ingestion were recorded between September 2017 and August 2018 at Alder Hey Children’s Hospital emergency department (ED). Of these, 70 (55.1%) were intentional overdoses (92.9% female), and 57 (44.9%) presenting with accidental overdose (54.4% female). 93% of accidental overdoses were under the age of 10 years. One hundred and 6 patients (83.4%) had paracetamol concentration measured on arrival to ED; 25 (19.7%) of these required treatment with N-acetylcysteine and 2 (1.6%) required vitamin K for abnormal blood clotting. Of those requiring treatment with N-acetyl cysteine 22 (17.3%) presented with intentional overdose and 3 (2.4%) presented with accidental ingestion. No patients were recorded as developing acute liver failure.^11^ The majority (n = 12, 9.4%) of those that did not have a paracetamol level measured had ingested <75 mg/kg, and therefore did not require further clinical intervention and 3 (2.4%) were >16 years of age and transferred to adult services. Zero (0%) patients had an undetectable paracetamol concentration.

The population of Liverpool in the 2011 UK Census was 466 415 of which 96 392 (21%) were aged 0 to 18 years.^12^ This gives a rate of 13.2 per 100 000 children per year presenting with paracetamol overdose in the Liverpool area. Extrapolating from our data it is estimated that in children alone there would be 16 492 cases presenting to EDs in England & Wales,^12^ of which approximately 7400 would be accidental overdoses.

## Discussion

The aim of this study was to examine the acceptability of a wearable device to help guide decision making in cases of suspected paracetamol overdose in children presenting to an ED. The use of wearable technology is of particular interest in paediatric healthcare where invasive blood sampling can be a traumatic experience. However little is known about how children, as well as their parents and healthcare professionals involved in their care, perceive their use. It is important to understand the acceptability of such a monitoring device to inform its design and potential implementation.

This study has demonstrated that amongst children, parents and health care professionals, a non-invasive device capable of measuring paracetamol concentration would be an acceptable and desirable innovation for use within paediatric populations, with an average of 81.5% of participants expressing a willingness to wear the device. There were several cautions expressed, which need to be considered in product development including the physical product dimensions amongst different age groups and the attractiveness of a device particularly amongst younger children who might struggle to wear the device for prolonged periods of time without significant distraction or placation. Reassuringly all of the older children and parents ranked accuracy of results as the most important feature of the device which again would help to avoid unnecessary blood sampling.

The main limitation of this study was the relatively small population size. We were also unable to capture information on concerns for wearing the device in the 12 to 15 years age group. Future studies that utilise the device for studies of accuracy will also be needed to ensure that developments in design and comfort continue to match expectations.

Although overall morbidity and mortality in young children that have taken accidental overdose is very low^13^ this is not the case in teenagers. Teenagers are more likely to present late and ingest several supratherapeutic doses, both factors are known to be associated with worse outcomes.^14^ Careful consideration would be needed before this device were considered in such ‘high risk’ populations, however acceptable the concept is to the teenage population. The avoidance of painful blood tests make it an obvious choice for younger children, who represent a more straightforward proposition for implementation given their lower risk. The positive responses from parents and healthcare professionals for the device and concept is also a useful finding from this work, and the next step would be a clinical study to ascertain the accuracy and validity of the device as part of a device development programme leading to CE mark accreditation.

The assessment of paracetamol overdose in children represents a relatively modest commercial opportunity. Based on the statistics for Liverpool and typical pricing for existing point of care testing, we estimate the total global market to be in the region of £10 m. It does however offer the potential for an early entry point for non-invasive testing into a clinical setting and the provision of benefits to the patient, the clinician and ED efficiency. As an example, as well as its use in paracetamol overdose, the device would also be of use in mixed overdoses, including those not thought to involve paracetamol ingestion with many hospitals routinely measuring paracetamol concentrations in all overdose patients.^15-17^ It will also increase awareness of non-invasive devices with the potential identification of further areas in which such devices could meet currently unmet clinical needs.

## Conclusion

Acceptability of a non-invasive paracetamol sensor was high in all groups, provided accuracy could be assured.

## Summary

- Paracetamol overdose is a common presentation to children’s emergency departments in the UK but diagnosis is dependent on time specific blood tests that are often distressing to children.
- A wearable device has been developed, designed to determine serial paracetamol concentration in sweat.
- This study has demonstrated that amongst children and teenagers, the majority would wear the device and see it as preferential to a blood test.
- The device needs to be made smaller and lighter but once these design aspects have been addressed it has the potential to avoid invasive blood sampling in children.

## Supplemental Material

sj-docx-1-bec-10.1177_11795972221140108 – Supplemental material for Assessing Paracetamol Overdose in Children: Acceptability and Potential Market for a Non-Invasive Testing Device Click here for additional data file. Supplemental material, sj-docx-1-bec-10.1177_11795972221140108 for Assessing Paracetamol Overdose in Children: Acceptability and Potential Market for a Non-Invasive Testing Device by Debora Freitas, Christopher Parry, Gabrielle Seddon, Jana Lemke, James Moss, Neville Freeman, Julie Grice and Daniel B Hawcutt in Biomedical Engineering and Computational Biology
