# Supplementary material for: Assessing Paracetamol Overdose in Children: Acceptability and Potential Market for a Non-Invasive Testing Device
Source: Biomed Eng Comput Biol. 2023 Jan 31;14:11795972221140108. doi: 10.1177/11795972221140108 (PMC9902896; doi:10.1177/11795972221140108)
Supplement: sj-docx-1-bec-10.1177_11795972221140108 – Supplemental material for Assessing Paracetamol Overdose in Children: Acceptability and Potential Market for a Non-Invasive Testing Device [file sj-docx-1-bec-10.1177_11795972221140108.docx]

Assessing paracetamol overdose in children: acceptability and potential market for a non-invasive testing device

(SUPPLEMENTARY DATA SECTION)

Debora Freitas^1^, Christopher Parry^2,3^, Gabrielle Seddon^4^, Jana Lemke^5^, James Moss^2,3^ Neville Freeman^6,7^ Julie Grice^1^, Daniel B Hawcutt^2,3^

Affiliations:

1: Emergency Department, Alder Hey Children’s Hospital, Liverpool, UK

2: NIHR Alder Hey Clinical Research Facility, Liverpool, UK

3: Department of Women’s and Children’s Health, Institute of Life Course and Medical Sciences, University of Liverpool, UK

4: Paediatric Medicines Research Unit, Alder Hey Children’s Hospital, Liverpool, UK

5: University of Greifswald, Germany

6: Nanoflex Ltd, Warrington, UK

7: E4G Ltd, Neston, UK

Corresponding Author:

Daniel B Hawcutt, University of Liverpool, Alder Hey Children’s Hospital, Eaton Road, Liverpool, L12 2AP. Email: Dhawcutt@liverpool.ac.uk Tel: 0044 1512284811

# Methods

The following age-appropriate questionnaires were distributed amongst the participants:

Participant ID: ___________

**PELICAN Study**


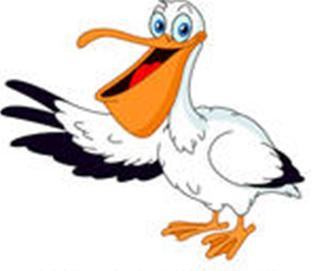


## **Questionnaire for 6-11 year olds**

*If you are happy to participate in the PELICAN (ParacEtamoL overdose In Children: Acceptability of Non-Invasive testing) Study, please complete this questionnaire.*

*If you do not wish to participate, please do not complete the questionnaire.*

*If you have any questions, please let a member of the research team know and they will help you.*

**Question 1**

How old are you? ________

**Question 2**

Are you a

- Boy
- Girl
- Prefer not to say

**Question 3**

Would you prefer to have a blood test or would you prefer to have paracetamol levels measured with a device that sits on your wrist?

- Blood Tests
- Device
- Don’t know/not sure

**Question 4**

This is a picture of the device:


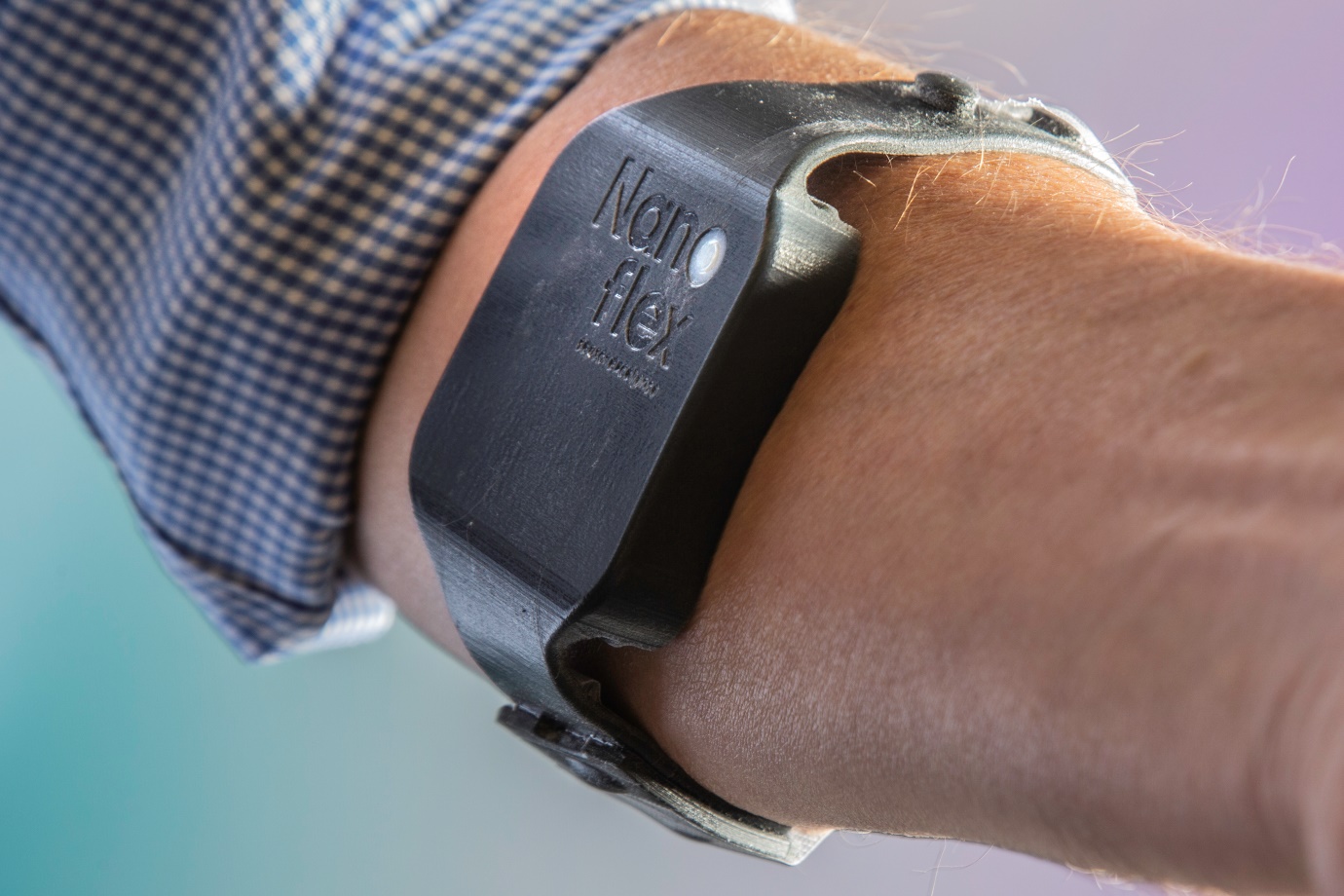


Do you like how it looks?

- Yes
- No

**Question 5**

If you could make the device any colour, what colour would you like it to be?

____________________

**Question 6**

What shape and style would you like the device to be?

*Please draw a picture below, or use words to describe how you would like it to look.*

**Question 7**

Would you be happy to wear this device on your wrist?

- Yes
- No

**Question 8**

How long would you be happy to wear this device for?

- Less than 30 minutes (1 cartoon episode)
- 30 minutes to 1 hour (lunch break at school)
- 1 hour to 2 hours (a film)
- 2 hours to 3 hours (the time between lunch and going home from school)
- 3 hours to 4 hours (the time between going to school and having your lunch)

**Question 9**

Would you have any worries about wearing the device?

- Yes
- No

If yes…

What would your worry be?

____________________________________________________________________________________________________________________________________________________

**Question 10**

Is there anything else you would like to tell us about the device?

______________________________________________________________________________________________________________________________________________________________________________________________________________________________

**Thank you for completing this questionnaire ☺!**

Participant ID: ____________

**PELICAN Study**


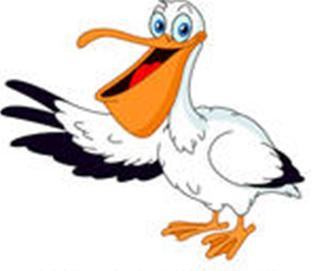


## **Questionnaire for 12-15 year olds**

*Please only complete this questionnaire if both you and your parent or guardian are happy for you to participate in the PELICAN (ParacEtamoL overdose In Children: Acceptability of Non-Invasive testing) Study.*

*If your parent/guardian is also happy to tell us their views on the acceptability of the non-invasive device, there is a parent-specific questionnaire that they can complete.*

*If you do not wish to participate, please* ***do not*** *complete the questionnaire.*

*If you have any questions, please let a member of the research team know and they will be happy to help you.*

**Question 1**

How old are you? ________

**Question 2**

Are you a

- Boy
- Girl
- Prefer not to say

**Question 3**

If there were worries that you may have had too much paracetamol, would you prefer to have an initial blood test(s) or for your paracetamol levels to be measured with a device that sits on your wrist?

- Blood Tests
- Device
- Don’t know/not sure

**Question 4**

This is a picture of the device:


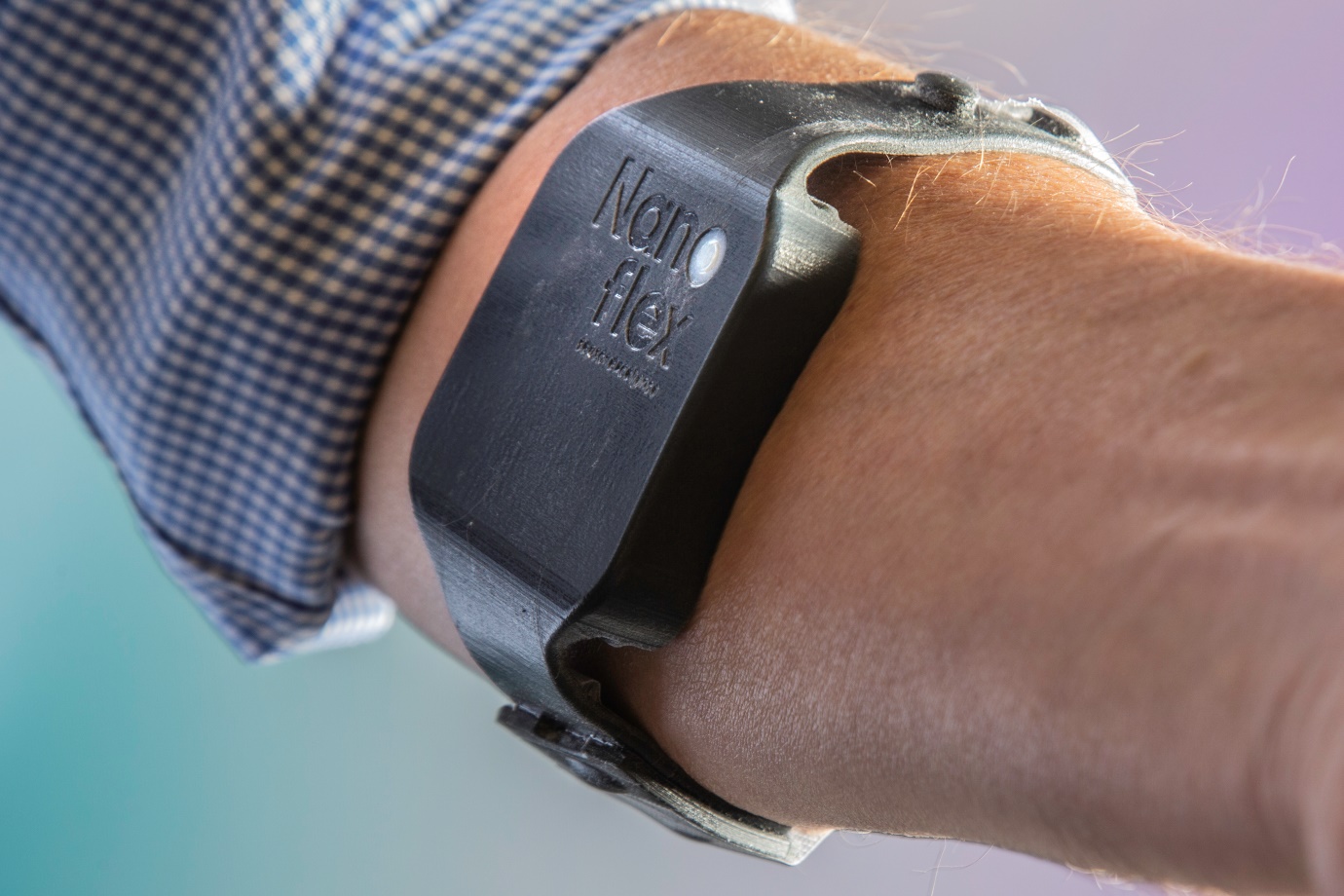


Do you like how it looks?

- Yes
- No

**Question 5**

If the device could be any shape, colour and style, how would you like it to look? Please describe this below.

______________________________________________________________________________________________________________________________________________________________________________________________________________________________

**Question 6**

How long would you be happy to wear the device for?

- Less than 30 minutes
- 30 minutes to 1 hour
- 1 hour to 2 hours
- 2 hours to 3 hours
- 3 hours to 4 hours

**Question 7**

If you had to wear the device, which of the following would be the most important?

Please score the following items in order of importance, where 1 is the most important and 4 is the least important.

- Avoidance of blood tests
- Comfort
- Accuracy
- Less time to treatment/Quicker diagnosis

We know that if blood paracetamol levels are too high, the liver can be damaged, so doctors use the results of these blood tests to decide who needs treatment.

If this device works as expected, then only children with high paracetamol readings on the device would need to have a blood test, and maybe treatment. This would mean that children who have low levels of paracetamol may avoid blood tests.

This device uses sweat not blood, to measure paracetamol levels.

The medical team would always use other information as well as the device to decide if the child needed a blood test – like the story and any other illnesses the child has.

There are two risks with a new way of measuring –

1. The device detects a high level of paracetamol in children who have low levels (resulting in unnecessary blood tests)

2. The device detects a low level of paracetamol in children who have high levels (risk of missing children with high levels)

**Question 8**

Which of the following would you be most concerned about?

- The device detects a high level of paracetamol in children who have low levels (resulting in unnecessary blood tests)
- The device detects a low level of paracetamol in children who have high levels (risk of missing children with high levels)
- Both equally important

**Question 9**

If there was a concern that the paracetamol levels in your blood were too high, would you be willing to take part in a clinical trial comparing the device with the standard blood test? This would not involve additional blood tests, but would test the safety and accuracy of the device.

- Yes
- No

**Question 10**

Is there anything else you would like to tell us about your views on the device?

_____________________________________________________________________________________________________________________________________________________________________________________________________________________________________________________________________________________________________________________________

**Thank you for completing this questionnaire ☺!**

Participant ID: __________

**PELICAN Study**


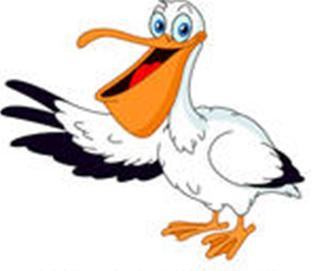


## **Questionnaire for Participants aged 16-18 years**

*If you are happy to participate in the PELICAN (ParacEtamoL overdose In Children: Acceptability of Non-Invasive testing) Study, please complete this questionnaire.*

*We are doing some research to find out what patients, families and healthcare professionals think of using a non-invasive device to measure paracetamol levels in children who may have taken a paracetamol overdose. By completing this questionnaire you will be able to tell us what you think and aid in the design of the device.*

*This questionnaire is designed for patients aged 16-18 years.*

*All your answers are confidential and will only be read by the team doing the research. We will put your answers together with answers from lots of other patients and their families and they will be summarised and shared with colleagues who work for Nanoflex who are developing the device.*

*If you do not wish to participate, please* ***do not*** *complete the questionnaire.*

*If you have any questions, please let a member of the research team know and they will be happy to help you.*

**ABOUT YOU**

**Question 1**

How old are you? _____

Are you

- Male
- Female
- Prefer not to say

**Question 2**

If there were worries that you may have had too much paracetamol, would you prefer to have an initial blood test(s) or for your paracetamol levels to be measured with a device that sits on your wrist?

- Blood Tests
- Device
- Don’t know/not sure

**ABOUT THE DEVICE**

**Question 3**

This is a picture of a prototype of the device:


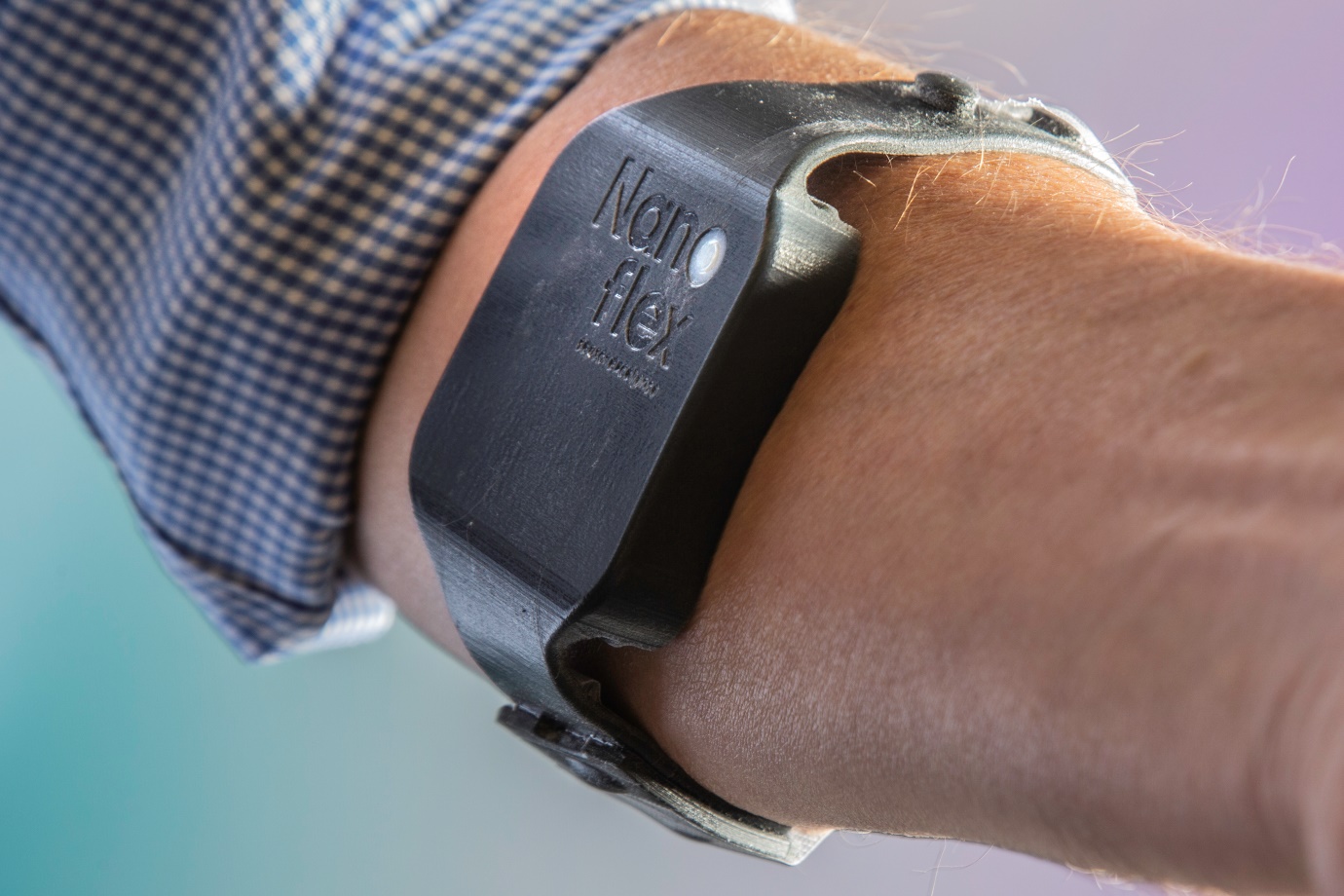


Do you think you would be happy to wear this?

- Yes
- No

**Question 4**

Do you envisage any problems with wearing this device?

- No
- Yes

If yes, what do you think the problems will be?

____________________________________________________________________________________________________________________________________________________________________________________________________________________________________________________________________________________________________________

**Question 5**

How long do you think you would tolerate wearing the device for?

- <0.5 hour
- 0.5 hour – 1 hour
- 1 hour – 2 hours
- 2 hours – 3 hours
- 3 hours – 4 hours

**Question 6**

If you had to wear the device, what would be the most important factor to you? Please score the following factors in order of importance, where most important is 1 and least important is 4.

- Avoidance of blood tests
- Comfort
- Accuracy
- Less time to treatment/Quicker diagnosis

We know that if blood paracetamol levels are too high, the liver can be damaged, so doctors use the results of these blood tests to decide who needs protective treatment.

If this device works as expected, then only those with high paracetamol readings on the device would go on to get a blood test, and maybe treatment. This would mean that children who have low levels of paracetamol can avoid unnecessary blood tests.

This device uses sweat not blood, to measure paracetamol levels.

The medical team would always use other information as well as the device to decide if the child needed a blood test – like the story and any other illnesses the child has.

There are two risks with a new way of measuring –

1. The device detects a high level of paracetamol in children who have low levels (resulting in unnecessary blood tests)

2. The device detects a low level of paracetamol in children who have high levels (risk of missing children with high levels)

**Question 7**

Which of the following would you be most concerned about?

- The device detects a high level of paracetamol in children who have low levels (resulting in unnecessary blood tests)
- The device detects a low level of paracetamol in children who have high levels (risk of missing children with high levels)
- Both equally important

**Question 8**

If there was a concern that the paracetamol levels in your blood were too high, would you be happy to take part in a clinical trial comparing the device with the standard blood test? This would not involve additional blood tests, but would test the accuracy and safety of the device.

- Yes
- No

**Question 9**

Is there anything else you would like to tell us about your views on the device?

____________________________________________________________________________________________________________________________________________________________________________________________________________________________________________________________________________________________________________

**Thank you for completing this questionnaire!**

Participant ID: ___________

**PELICAN Study**


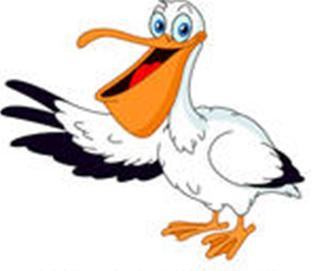


## **Questionnaire for Healthcare Professionals**

*If you are happy to participate in the PELICAN (ParacEtamoL overdose In Children: Acceptability of Non-Invasive testing) Study, please complete this questionnaire.*

*We are doing some research to find out what patients, families and healthcare professionals think of using a non-invasive device to measure paracetamol levels in children who may have taken a paracetamol overdose. By completing this questionnaire you will be able to tell us what you think and aid in the design of the device.*

*This questionnaire is designed to be completed by Healthcare Professionals who care for children.*

*All your answers are confidential and will only be read by the team doing the research. We will put your answers together with answers from lots of other families and they will be summarised and shared with colleagues who work for Nanoflex who are developing the device.*

*If you do not wish to participate, please* ***do not*** *complete the questionnaire.*

*If you have any questions, please let a member of the research team know and they will help you.*

***Question 1***

What is your job role?

- Doctor
- Nurse
- Pharmacist
- Health Care Assistant
- Other – Please specify ___________________

***Question 2***

Do you think it is a good idea to introduce a non-invasive device to measure paracetamol levels?

- Yes
- No
- Don’t know/not sure

***Question 3***

This is a picture of a prototype of the device:


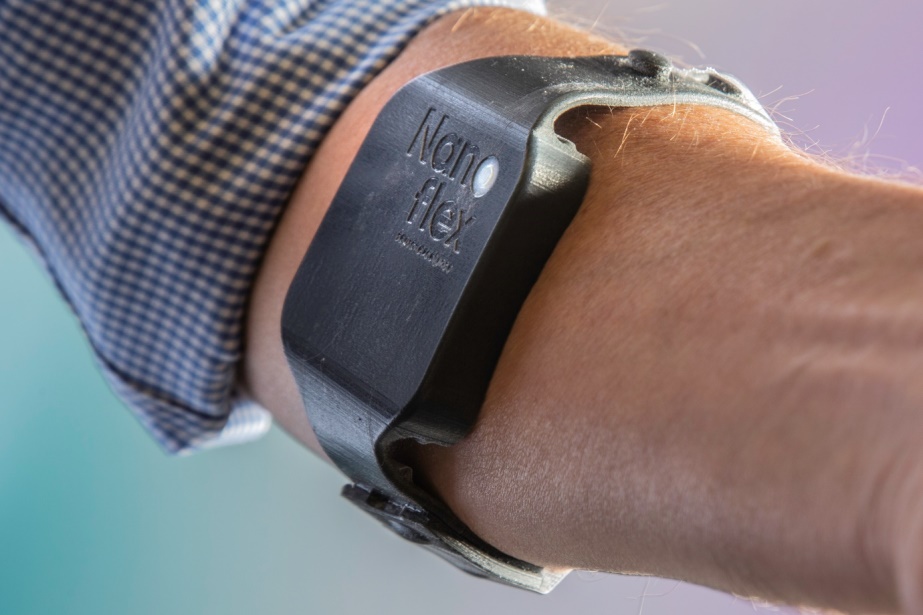


Do you think children would be happy to wear this?

- Yes
- No

***Question 4***

Do you envisage any problems with children wearing this device?

- No
- Yes

If yes, what do you think the problems will be?

____________________________________________________________________________________________________________________________________________________________________________________________________________________________________________________________________________________________________________

As you know, if blood paracetamol levels are too high, the liver can be damaged, so the medical team use the blood test results to decide who needs protective treatment.

If this device works as expected, then only those with high paracetamol readings on the device would go on to get a blood test, and maybe treatment. This would mean that children who have low levels of paracetamol can avoid unnecessary blood tests.

This device uses sweat to measure paracetamol levels, rather than blood.

There are two risks with a new way of measuring –

1. The device detects a high level of paracetamol in children who have low levels (resulting in unnecessary blood tests)

2. The device detects a low level of paracetamol in children who have high levels (risk of missing children with high levels)

***Question 5***

Select the statement below that you perceive to be the greatest risk.

- The device detects a high level of paracetamol in children who have low levels (resulting in unnecessary blood tests)
- The device detects a low level of paracetamol in children who have high levels (risk of missing children with high levels)
- Both equally important.

***Question 6***

Which of the following features of the device would you rate as most important for a **low risk child**. Please score the following factors in order of importance, where most important is 1 and least important is 4.

- Tracking of paracetamol concentrations
- Rapid initial reading
- Further data in less than 4 hours
- Trend of paracetamol concentration

***Question 7***

Which of the following features of the device would you rate as most important for a **high risk child**. Please score the following factors in order of importance, where most important is 1 and least important is 4.

- Tracking of paracetamol concentrations
- Rapid initial reading
- Further data in less than 4 hours
- Trend of paracetamol concentration

***Question 8***

Would you be prepared to be involved in a clinical trial comparing the device with the standard blood test? This would not involve additional blood tests for the children you were caring for, but would test the accuracy and safety of the device.

- Yes
- No

***Question 9***

Would you be willing to use this device in clinical practice?

- Yes
- Yes – but only after a clinical trial
- No
- Unsure

***Question 10***

Is there anything else you would like to tell us about your views on the device?

____________________________________________________________________________________________________________________________________________________________________________________________________________________________________________________________________________________________________________

Participant ID:__________

**PELICAN Study**


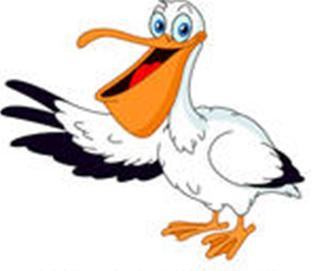


## **Questionnaire for Parents**

*If you are happy to participate in the PELICAN (ParacEtamoL overdose In Children: Acceptability of Non-Invasive testing) Study, please complete this questionnaire.*

*This questionnaire should be filled in by a parent or guardian but you are welcome to discuss it with your child and to add their comments.*

*We are doing some research to find out what patients, families and healthcare professionals think of using a non-invasive device to measure paracetamol levels in children who may have taken a paracetamol overdose. By completing this questionnaire you will be able to tell us what you think and aid in the design of the device.*

*All your answers are confidential and will only be read by the team doing the research. We will put your answers together with answers from lots of other families and they will be summarised and shared with colleagues who work for Nanoflex who are developing the device.*

*If you do not wish to participate, please* ***do not*** *complete the questionnaire.*

*If you have any questions, please let a member of the research team know and they will help you.*

**ABOUT YOUR CHILD**

**Question 1**

How old is the child that you are here with today? _____

Is the child you are here with today a

- Boy
- Girl
- Prefer not to say

**Question 2**

Do you have any other children?

- No
- Yes

If yes, please tell us the age and gender of each child:

Age: _____ Gender: Boy/Girl/Prefer not to say (please circle)

Age: _____ Gender: Boy/Girl/Prefer not to say (please circle)

Age: _____ Gender: Boy/Girl/Prefer not to say (please circle)

Age: _____ Gender: Boy/Girl/Prefer not to say (please circle)

**Question 3**

If there were worries that the child you are here with today may have had had too much paracetamol, would you prefer for them to have an initial blood test(s) or for their paracetamol levels to be measured with a device that sits on their wrist?

- Blood Tests
- Device
- Don’t know/not sure

**ABOUT THE DEVICE**

**Question 4**

This is a picture of a prototype of the device:


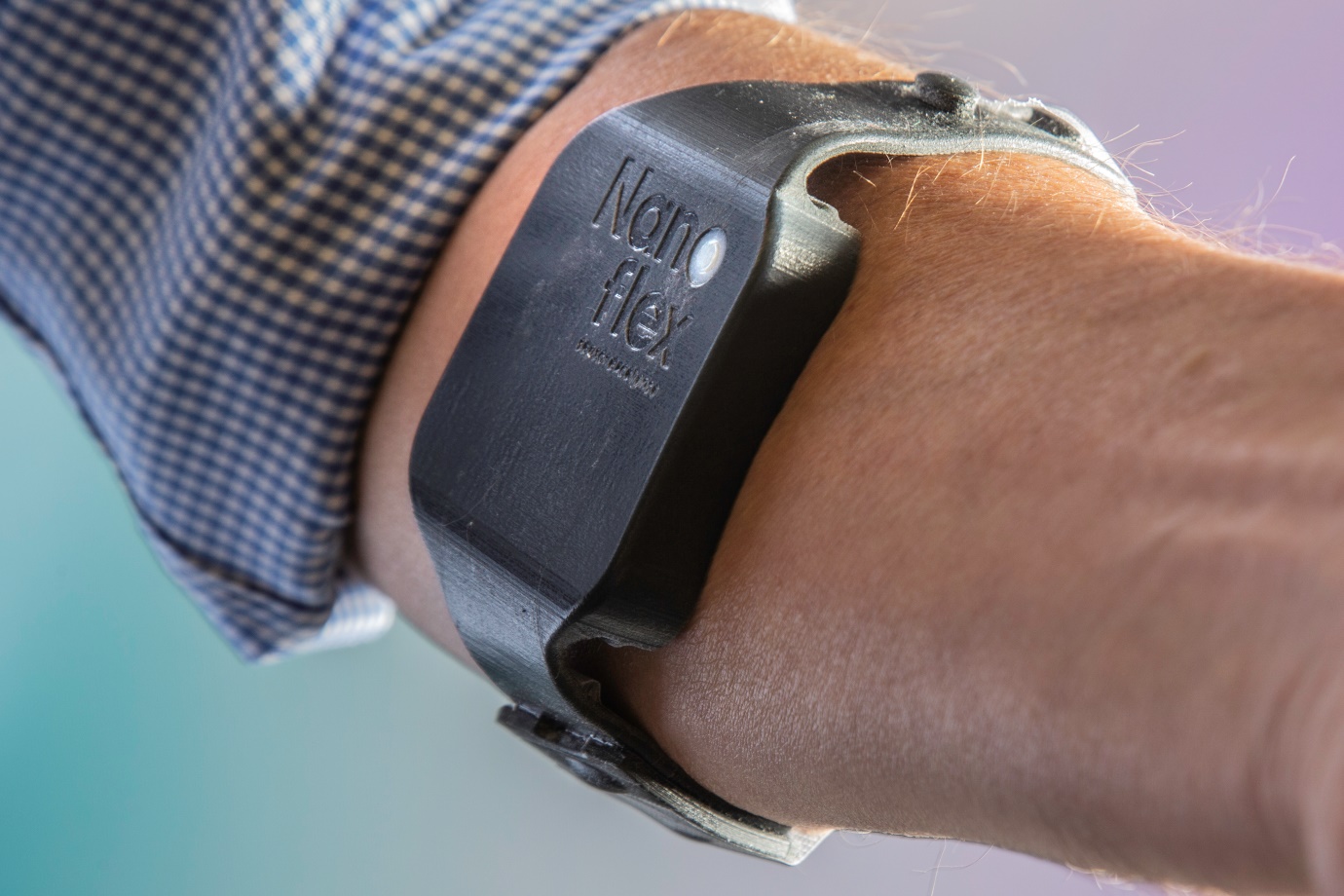


Do you think your child would be happy to wear this?

- Yes
- No

**Question 5**

Do you envisage any problems with your child wearing this device?

- No
- Yes

If yes, what do you think the problems will be?

____________________________________________________________________________________________________________________________________________________________________________________________________________________________________________________________________________________________________________

**Question 6**

How long do you think your child would tolerate wearing the device for?

- <0.5 hour
- 0.5 hour – 1 hour
- 1 hour – 2 hours
- 2 hours – 3 hours
- 3 hours – 4 hours

**Question 7**

If your child had to wear the device, what would be the most important factor to you? Please score the following factors in order of importance, where most important is 1 and least important is 4.

- Avoidance of blood tests
- Comfort
- Accuracy
- Less time to treatment/Quicker diagnosis

We know that if blood paracetamol levels are too high, the liver can be damaged, so doctors use the results of these blood tests to decide who needs protective treatment.

If this device works as expected, then only those with high paracetamol readings on the device would go on to get a blood test, and maybe treatment. This would mean that children who have low levels of paracetamol can avoid unnecessary blood tests.

This device uses sweat not blood, to measure paracetamol levels.

The medical team would always use other information as well as the device to decide if the child needed a blood test – like the story and any other illnesses the child has.

There are two risks with a new way of measuring –

1. The device detects a high level of paracetamol in children who have low levels (resulting in unnecessary blood tests)

2. The device detects a low level of paracetamol in children who have high levels (risk of missing children with high levels)

**Question 8**

Which of the following would you be most concerned about?

- The device detects a high level of paracetamol in children who have low levels (resulting in unnecessary blood tests)
- The device detects a low level of paracetamol in children who have high levels (risk of missing children with high levels)
- Both equally important

**Question 9**

If there was a concern that the paracetamol levels in your child’s blood were too high, would you allow your child to take part in a clinical trial comparing the device with the standard blood test? This would not involve additional blood tests, but would test the accuracy and safety of the device.

- Yes
- No

**Question 10**

Is there anything else you would like to tell us about your views on the device?

____________________________________________________________________________________________________________________________________________________________________________________________________________________________________________________________________________________________________________

**Thank you for completing this questionnaire!**
